# Sustainability status, sensitive and key factors for increasing rice production: A case study in West Java, Indonesia

**DOI:** 10.1371/journal.pone.0274689

**Published:** 2022-12-30

**Authors:** Benny Rachman, Ening Ariningsih, Tahlim Sudaryanto, Mewa Ariani, Kartika Sari Septanti, Cut Rabiatul Adawiyah, Ashari, Adang Agustian, Handewi Purwati Saliem, Herlina Tarigan, Syahyuti, Erny Yuniarti

**Affiliations:** 1 Research Center for Behavioral and Circular Economics–National Research and Innovation Agency, Jakarta, Indonesia; 2 Indonesian Center for Agricultural Socio Economics and Policy Studies–Ministry of Agriculture, Bogor, Indonesia; 3 Research Center for Social Welfare, Village, and Connectivity–National Research and Innovation Agency, Jakarta, Indonesia; 4 Research Center for Cooperative, Corporation, and People’s Economy–National Research and Innovation Agency, Jakarta, Indonesia; 5 Research Center for Horticultural and Estate Crops–National Research and Innovation Agency, Jakarta, Indonesia; ICAR-National Rice Research Institute, INDIA

## Abstract

The Indonesian rice production balance has managed to show a slightly consistent surplus recently, in the period of 2010 to 2021, but the country has continued to import rice to secure its rice reserve. The country has also made some efforts to increase its domestic rice production and, at the same time, faced ecological, socio-cultural, economic, institutional, and technological sustainability challenges. Previous studies on rice sustainability have shown varied results on the sustainability statuses and sensitive factors in Indonesia, yet there have been limited studies identifying key factors systematically. To provide more solid empirical evidence on this subject, a study to expand the scope to other sites with an additional analysis of the key factors is perceivably needed. This study aims to verify the sustainability status and identify sensitive factors as well as key factors for increasing rice production. The primary data were collected by interviewing officials from various agencies at the central and regional levels and several discussion groups of 40 participants, including farmers. In addition, secondary data were also collected from various ministries/agencies at the central and regional levels. Data analyses use a set of indicators, i.e., the Multidimensional Scaling (MDS) approach and the Matrix of Cross Impact Multiplications Applied to Classification (MICMAC) approach. The results show that the multidimensional sustainability status of increasing rice production in Bandung district is moderate, whereas sustainability status per dimension shows variations from poor to moderate. Fourteen out of 50 attributes are identified as sensitive factors influencing the sustainability of rice production. Six key factors are found to influence the sustainability of rice production. The study concludes that the sustainability status of increasing rice production in Bandung district is moderate, with variation across dimensions. The sustainability of increasing rice production in the study site is influenced by those 14 sensitive factors and those six key factors. This study recommends a number of major policies/programs to increase the sustainability of rice production, which are as follows: implementation of the Regional Spatial Plan, promotion of the application of best practices of farming management (organic fertilizers and pesticides), promotion of the use of agricultural machinery, and provision of farmer assistance for pre-harvest and postharvest production facilities, as well as farm financing. Future research should expand study sites to some other rice production centers with different attributes to enrich our understanding of the subject of rice sustainability.

## Introduction

Rice is a quasi-public good for the Indonesian people, which has strategic value in terms of economy, environment, social, and politics [1–3]. The demand for rice continues to increase along with the increasing population and the Indonesian economy. The recent data show that rice consumption increased steadily at 1.21% per annum, whereas rice production tended to stay stagnant at a growth rate of less than 1% per annum [4]. Even though the rice production balance showed a consistent surplus, Indonesia needs to continue to import rice to secure its rice reserves. The stagnant growth trend of rice production in Indonesia from 2010 to 2022 was due to limited or low extensification of farmland, a saturation of farming technology, and competition with high-value commodities [5]. To meet and anticipate the growing demand for rice, hence, the government has strived to both maintain and increase its domestic rice production through extensification, intensification, land optimization, reducing yield losses, and improving irrigation infrastructures.

However, such efforts to increase rice production can also bring a number of aspects on sustainability issues to deal with, one of which is from ecological aspects [6]. The main problems related to ecological aspects are the limited farming land and the degraded land along with the impact of climate change and unsustainable farming practices [7]. The significance of sustainable farming practice is clearly identified here. The other issues faced are from the aspects of socio cultural, economic, institutional, and technological.

Ghelichkhan et al. [8] defined sustainability as an effort to meet current food needs without forgetting the food needs of future generations. Furthermore, economic pillars for survival, environmental responsibility, and social acceptance can be developed to increase public understanding of the significance of sustainability [8, 9]. According to Chivenge et al. [10], applying a site-specific nutrient management technique can promote sustainability in rice by increasing rice yields, profit, and N usage efficiency while decreasing N losses. Meanwhile, Nadir et al. [11] claimed that the goal of sustainable rice production could be achieved by combining the disciplines of genomics, breeding, and weed management. In addition, they mentioned that enhancing rice producers’ knowledge, skills, and social networks, as well as improving land productivity and applying integrated nutrient management, can boost sustainable rice production.

The massive use of inorganic fertilizers, pesticides, and the intensive exploitation of land in the long-term result in environmental degradation, including soil structure. This leads to agricultural land conditions becoming increasingly critical [12–14]. In irrigated paddy fields, land damage occurs due to salinity, imbalanced availability of macro-micro nutrients, poor soil drainage and aeration, low soil organic matter content, and ecological imbalance of flora and fauna in paddy fields [15, 16].

Efforts to fulfill the basic needs for food in Indonesia are not always easy to make. Indeed, it is true, considering Indonesia’s large population reaching 270.2 million in 2020 [17], and is predicted to increase to 319 million by 2045 [18]. On top of that, future agricultural development generally faces increasingly limited resources, polluted and degraded land and water, conversion of agricultural land, and climate change [19], whereas land health and water supply are the most valuable resources for food security [20]. Not surprisingly, the importance of this concept of a sustainable agricultural system has been stated in the Sustainable Development Goals (SDGs).

As for the Indonesian context, some studies have analyzed the sustainability of rice in terms of rice cultivation/farming [21–28], paddy fields [29, 30], rice agribusiness [31], and rice availability [32–34] in some regions of the country. The studies show different results among the areas, showing sustainability statuses vary from less (poor) to moderately sustainable. Furthermore, the studies reveal various indicators that serve as the key factors for sustainability, showing specific locations [35]. In short, previous studies have concluded the sustainability status and sensitive factors in various locations, but not many studies analyze and focus on the key factors for increasing rice production.

The main objective of this study is (1) to verify the sustainability status of increasing rice production and (2) to identify sensitive factors and key factors for increasing rice production. The study contributes to a better understanding of sustainability in rice production by a deeper analysis of sensitive factors and key factors with a slightly different analytical method. Since sustainability status varies across sites, this study also wishes to add more evidence from different rice production centers.

## Methods

### Study area

Bandung district, West Java province, was selected as the study site based on the following considerations: (1) the area represents a rice production center with lowland agroecosystems, with rice production reaching 277.16 thousand tons of milled dry grains in 2020, (2) there is a high conflict of interest in land use, and (3) the area is part of the Upper Saguling Watershed confronted with soil and water pollution problems and a degradation problem of paddy fields. The fact that the area is next to Bandung city, the capital of West Java province, it potentially leads to a high degree of land conversion.

### Data collection

This study, conducted between May to September 2020, used primary and secondary data. The primary data and information collected were mainly related to the dimensions and attributes of sustainable efforts in increasing rice production. Respondents of the primary data include (1) relevant and competent institutional leaders/staff at the central level, namely the Directorate General of Food Crops, Directorate General of Horticulture, Food Security Agency, Directorate General of Agricultural Infrastructure and Facilities, and Research and Development Center, Ministry of National Development Planning, BPS-Statistics Indonesia (2) relevant and competent institutional leadership/staff at the selected provincial and district level: Office of Food Crops and Horticulture, Office of Food Security, Regional Development Planning Agency, and Office of Public Works and Spatial Planning; (3) 40 respondents in discussion groups, wherein each discussion group there are farmer groups and key informants. The data from the respondents were collected by using a structured questionnaire with a survey method. The respondents’ responses are on a scale of 0 to 4. The secondary data were obtained from databases of related institutions at the central and regional levels.

### Methods of analysis

The Multidimensional Scaling (MDS) method was used to assess the sustainability status of rice production, whereas leverage analysis was used to analyze the sensitive factors for increasing rice production. Furthermore, the Matrix of Cross Impact Multiplications Applied to Classification (MICMAC) approach was employed to determine the key factors for a sustainable increase in rice production.

The analysis stages of sustainable rice production improvement are as follows: (1) reviewing the attributes in each dimension of sustainability and assessing these attributes; (2) scoring the attributes of each dimension of sustainability; (3) making the analysis of MDS with SPSS software to determine ordinance, sustainability index value, and stress value through ALSCAL; (4) assessing the sustainability index and status of increasing rice production, multidimensionally and each dimension, where the value of the sustainability score for each dimension is grouped into the following index interval and sustainability status [32]: 0.00‒25.00 (bad); 25.01‒50.00 (poor); 50.01‒75.00 (moderate); 75.01‒100.00 (good); (5) making the Monte Carlo analysis to analyze aspects of uncertainty [36, 37]; (6) conducting the sensitivity analysis to determine sensitive variables affecting sustainability; and (7) making participatory prospective analysis to determine the key factors influencing the increase in rice production using MICMAC [38].

#### Multidimensional Scaling (MDS)

The sustainability status of rice production was analyzed using the Rap-rice approach modified from the Rapfish Technique (Rapid Assessment Techniques for Fisheries) developed by Pitcher et al. [39]. Rap-rice is based on MDS’s ordinance technique [36, 40–42]. MDS is a statistical analysis technique that transforms each dimension and is multidimensional on the sustainability dimension [43]. Dimensions, attributes, and scoring were used to analyze the sustainability of increasing rice production [44]. The use of MDS in this study is based on the fact that this method produces stable parameter approximations [36]. Besides rice farming, MDS analysis is widely applied in various fields of agriculture such as corn production [45], palm oil [46, 47], corporate-based shallot farming business [48], integration of beef cattle and paddy farming [49], paddy fields [29], sugar industry [50], rural agropolitan [51], forestry community [52], and peatland [48, 53].

In this study, the sustainability analysis of rice production comprises five dimensions and 50 attributes, namely ecological (13 attributes), economic (13 attributes), social (11 attributes), institutional (5 attributes), and technological/infrastructure (8 attributes) (Table 1). In more detail, the 50 attributes that are distributed into five dimensions and the criteria for scoring are presented in S1 Appendix.

**Table 1:** Dimensions and attributes used in Rap-rice.

No.	Dimension				
	Ecological	Economic	Social	Institutional	Technological
**1**	Use of chemical fertilizers	Economic efficiency	Proportion of rice farm HHs	Farmers’ participation in farmer group activities	Number of tractors in the area (district)
**2**	Use of organic fertilizers	Rice farming profit	Farmers’ formal education	Presence of agricultural extension	Number of water pumps
**3**	Use of chemical pesticides	Rice production	Farmers’ participation in agricultural extension	Consistency of land use with RTRW (Regional Spatial Plan)	Number of threshers
**4**	Use of natural pesticides	Farmers’ exchange rate	Farmers’ motivation	Perpetual land status for rice (local government regulation)	Number of dryers
**5**	Rainfall	Rice price	Conflict in paddy field use	Proportion of local government budget for food crops sub-sector	Number of RMUs
**6**	Dry month	Real wages	Proportion of profit sharing	-	Farmers’ adoption of new high-yielding varieties
**7**	Irrigation system	Proportion of smallholder farmers	Family participation in rice farming	-	Farmers’ adoption of the jajar legowo system
**8**	Rice productivity	Agricultural workers	Rice consumption	-	Implementation of postharvest technology
**9**	Land conversion	Access to capital	Alternative business	-	-
**10**	New paddy field construction	Income from rice farming	Perceptions of rice farming sustainability	-	-
**11**	Crop failure	Marketing agency	Rice farming management pattern	-	-
**12**	Pressure on land use	Rice competitiveness	-	-	-
**13**	Rice waste utilization	Proportion of farmers with crop insurance	-	-	-

In MDS, the point of the observed object is mapped into two or three-dimensional space to attempt to approach the original object. The ordination technique (distance determination) in MDS is based on the squared Euclidean distance, which in n-dimensional space can be written as follows [54].

$$d_{ij}^{2}=\sum {\left(x_{ij}-x_{j}\right)}^{2}$$ (1)

where $d_{ij}^{2}$ is squared Euclidean distance, *x*_*ij*_ is attribute score values, *x*_*j*_ is average attribute score values, *i* is 1, 2, …, n, *j* is 1, 2, …, p.

The ordinance of an object point in MDS is approximated by regressing the Euclidean distance (*d*_*ij*_) from point *i* to point *j*, with the origin point (*d*_*ij*_) as in the following equation.

$$\hat{d_{ij}}=\alpha +{\beta d}_{ij}+e$$ (2)

where $\hat{d_{ij}}$ is estimated value, and *e* is error.

The approach techniques commonly used to regress the above equations are (1) the least square method and (2) the alternating least-squares algorithm (ALSCAL) method based on the roots of the Euclidian distance (square distance), and (3) the maximum likelihood method. In MDS, the ALSCAL algorithm is the most suitable method to be applied through statistical software/SPSS [32, 55, 56].

The ALSCAL method optimizes the squared distance to the squared data (origin = *dij*), which in three dimensions is written in a formula called S-stress as follows [54]:

$$S={\left\{\frac{\left(\sum_{i}\sum_{j}{\left(d_{ij}-\hat{d_{ij}}\right)}^{2}\right)}{{\left(\sum_{i}\sum_{j}(d_{ij}\right)}^{2}}\right\}}^{\frac{1}{2}}$$ (3)

Furthermore, in the MDS, a goodness of fit test needs to be carried out to measure how accurately the configuration of a point can reflect the original data. This goodness of fit in the MDS is reflected in the magnitude of the S-stress value. A low S-stress value indicates a fit model, while a high S-stress value indicates the opposite. A fit model is indicated by an S-stress value of less than 0.25 (S < 0.25).

#### Matrix of Cross Impact Multiplications Applied to Classification (MICMAC)

The MICMAC approach [57] was used to determine the key factors that influence rice production, with the following stages: (1) collecting important factors obtained from leverage analysis for evaluation by experts, and (2) filling in the matrix of direct influence (MDI) by experts, by quantifying the relationship between variables [58] with a scale of 0 to 3, meaning: (0) there is no relationship (no influence), (1) weak influence, (2) moderate influence, and (3) strong influence. Such data processing to determine key factors by MICMAC software has been widely used in various studies to determine sustainability variables [59–67].

MICMAC is a structural analysis tool that maps variables into influence and dependence components of variables within a system [67]. MICMAC analysis is used for variable mapping and determination of the main variable/key variable [68]. It is based on the multiplication property of the matrix. MICMAC excels at capturing variable interactions and identifying critical variables that can be used as a driver for a system to operate sustainably [69]. This modeling technique is often used to assist policy strategy planning, especially in identifying and concluding various relationships between factors in a particular problem or issue. The variables in the MICMAC analysis are arranged into four clusters, namely independent variables, dependent variables, linkage variables, and autonomous variables.

**Fig 1:**
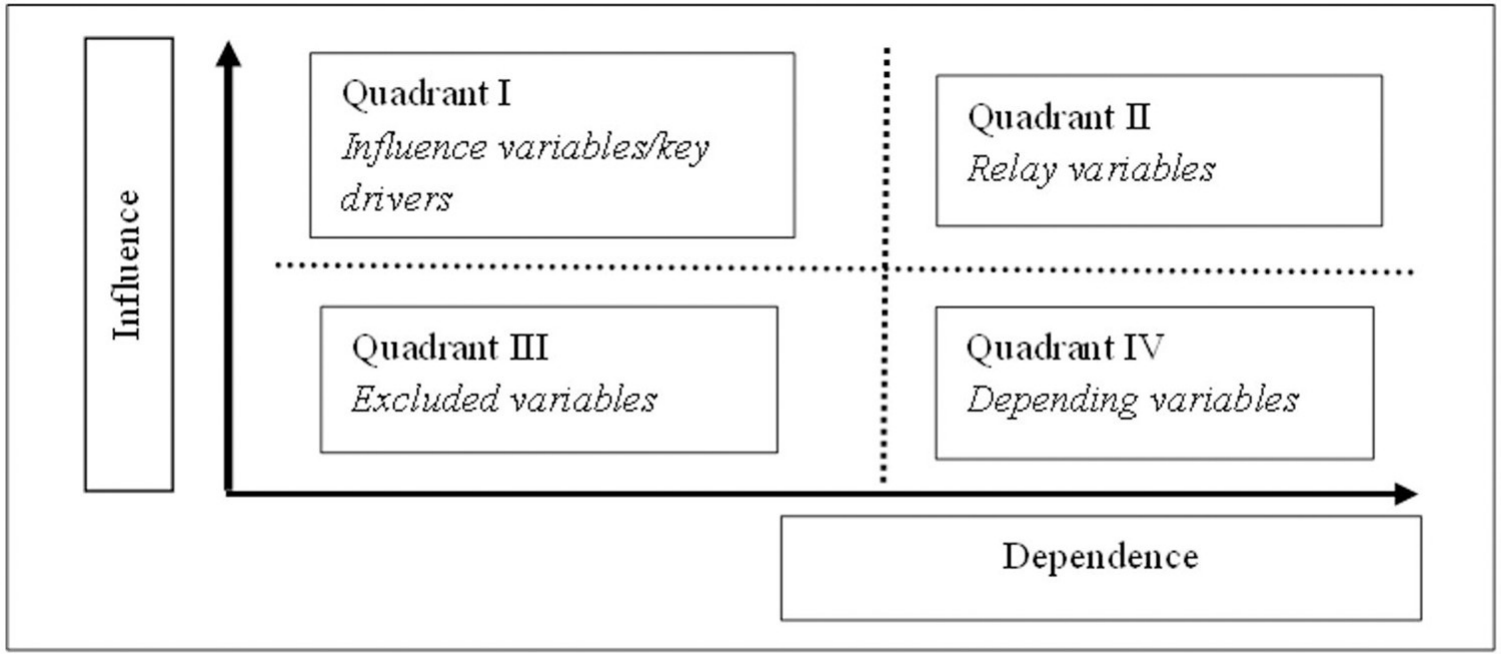
Influence and dependence factors.

The analysis results using the MICMAC method will then group the variables into four quadrants [70] (Fig 1). Quadrant I contains the variables very influential with little dependence on variables in other quadrants. Variables in this quadrant are key factors in the system. Quadrant II contains variables influential but highly dependent, which can cause instability in the system. Quadrant III contains the variables that are highly dependent but have little effect, so these variables are quite sensitive to changes in variables in quadrants 1 and 2. Quadrant IV describes variables that have a small influence and small dependence, so variables in this quadrant will not prevent a system from functioning [70].

## Results and discussion

### Sustainability status of rice production improvement

The sustainable management of the environment and its inherent resources is deemed one of the most severe challenges in the twenty-first century [71]. The concept of sustainable development strives to balance economic growth (economic dimension), environmental preservation (ecological dimension), and equity (socio-cultural dimension). In line with the developing dynamics, the sustainability aspect needs to consider other dimensions, namely the technological and infrastructure dimensions as well as the institutional dimension. Since its inception, such a notion has undergone several stages of development, adjusting to the changing needs of a global world. The core concepts and aspirations, as well as the difficulties in putting them into practice, remain largely constant, though [72].

Based on the multidimensional Rap-rice analysis results using the MDS ordinance, the sustainability status of rice production in Bandung district is categorically at a moderate level, with an index value of 50.57 (Table 2). Further examination of each factor reveals differences in the status of sustainability as described in its index value. Similarly, both the ecological and also technological dimensions are moderately sustainable, with index values of 50.57 and 61.76, respectively. In contrast, the sustainability status of the economic, social, and institutional dimensions are categorized as less sustainable, with index values of 35.25, 30.79, and 48.11, respectively.

**Table 2:** Statistical parameter, index, and sustainability status of rice production increase per dimension in Bandung district.

Dimension	MDS index	Monte Carlo	Delta	Stress	R^2^	Status^a^
**Ecological**	50.57	50.42	0.13	0.13	0.95	Moderate
**Economic**	35.25	36.57	-1.32	0.13	0.95	Poor
**Social**	30.79	32.26	-1.47	0.13	0.95	Poor
**Institutional**	48.11	47.74	0.36	0.14	0.93	Poor
**Technological**	61.76	60.52	1.23	0.13	0.95	Moderate
**Multidimension**	50.08	50.04	0.03	0.12	0.95	Moderate

^a^Note: 0.00‒25.00 (bad); 25.01‒50.00 (poor); 50.01‒75.00 (moderate); 75.01‒100.00 (good) [32].

Some previous studies have revealed lower sustainability indices of rice production than that in the research area, such as on marginal peat soils in Bengkulu (47.81) [21] and the wetland in Jambi district (41.96) [22]. Meanwhile, lowland rice farming in Subak Intaran Barat, Denpasar, has a sustainability index value of 73.48 or is moderately sustainable [28].

The sustainability of rice farming is closely related to the sustainability of paddy fields. The study result by Nurwadjedi et al. [30] reported that the agroecosystem zones of paddy fields in East Java province have different sustainability statuses. However, most of them are categorized as moderately sustainable. Threats to the sustainability of paddy fields are mainly caused by land conversion, land fragmentation, and aging farmers. Meanwhile, the sustainability status of paddy fields in Setianagara village, Cibeureum subdistrict, Tasikmalaya city, is poor multidimensionally and for each dimension analyzed (social, economy, ecology, technology, and law and institutional) [29].

**Fig 2:**
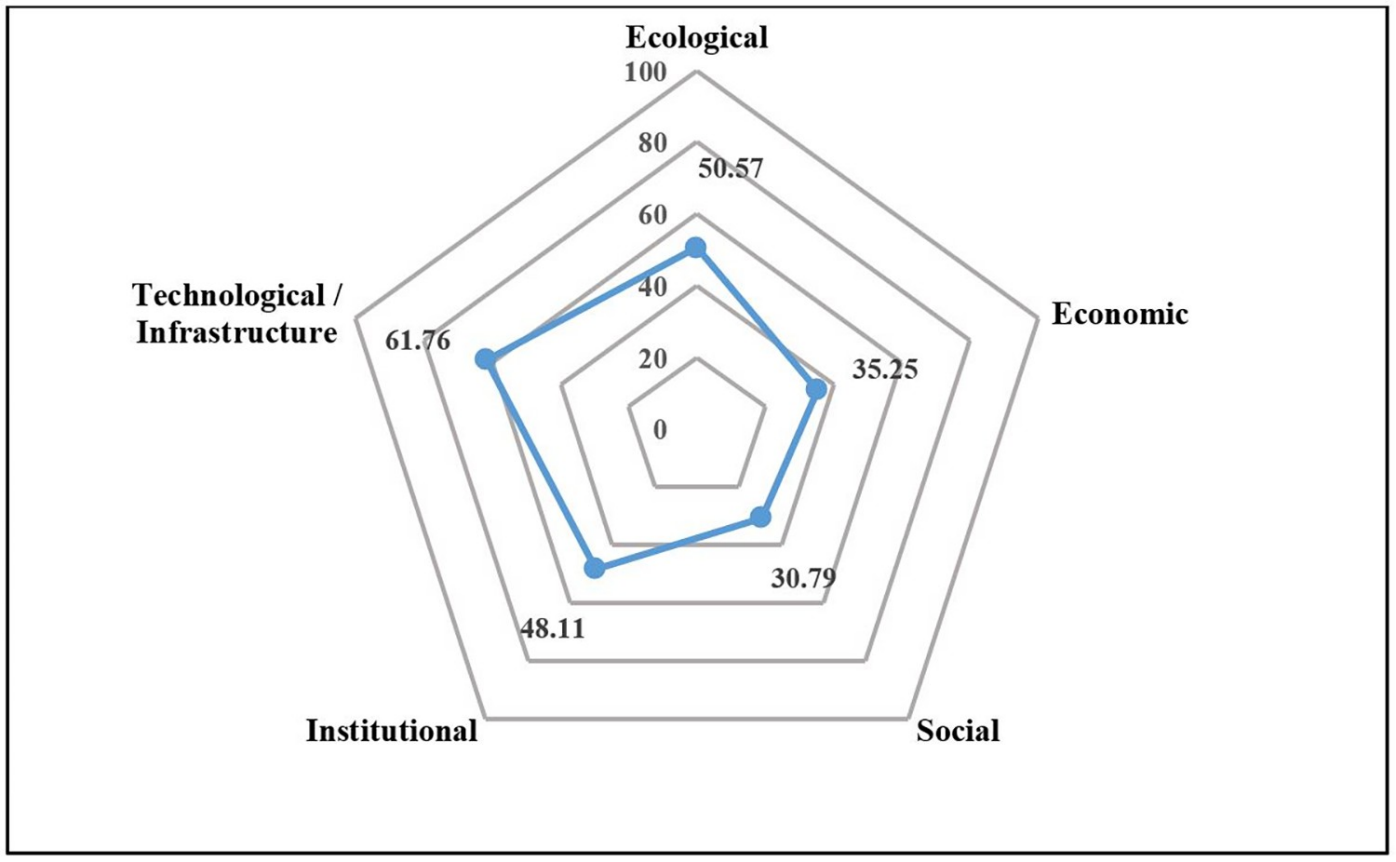
Kite diagram of the sustainability index of increasing rice production in Bandung district.

The results of the Monte Carlo analysis at the 95% confidence level showed no significant difference between the results of the MDS analysis in Bandung district and the results of the Monte Carlo test. This shows that the error in the analysis is very small. In addition, the results of the Rap-rice analysis show that all attributes provide accurate and accountable analysis results. This can be seen from the stress value, only around 0.13, and the coefficient of determination (R^2^) is 0.95. This value follows the opinion of Kavanagh et al. [73], which states that the analysis results are adequate if the stress value is less than 0.25 and the coefficient of determination (R^2^) is close to 1. A diagram depicting the sustainability status of increasing rice production in Bandung district for each dimension is presented in Fig 2.

Fig 2 shows that the technological/infrastructure dimension has the highest sustainability index among the five dimensions analyzed. In contrast, the social dimension has the lowest. The detailed discussion is elaborated in the following sections.

#### Ecological dimension

The results of the Rap-Rice analysis show that according to the ecological dimension, the sustainability status of increasing rice production in Bandung district is in the moderately sustainable category with an index value of 50.57. This is quite reasonable considering the two main reasons, namely the massive pest control activities carried out by the Plant Protection Brigade in collaboration with POPT and the PPL to support the sustainability of rice production in Bandung district. Pest control is carried out through spot stop and IPM movements. In addition, organic farming has begun to develop so that farmers have started to reduce the application of chemical pesticides and fertilizers. On the other hand, the sustainability of rice production in Bandung district also faces an obvious problem of high conversion of agricultural land (paddy fields) to non-agriculture, especially for industrial estates, infrastructures, and housing/settlements. Land conversion in Bandung district, especially in the peri-urban area, is difficult to prevent given the region’s rapid development. According to Rondhi et al. [74], housing in urban areas is worth seven times more than agricultural land. Furthermore, it has commonly been demonstrated that the price of agricultural land can go higher after conversion.

As a comparison regarding the ecological dimension, Yusuf et al. [25] showed that wetland rice farming in Siak district, Riau province, was in poor to moderate categories for the four subdistricts analyzed (sustainability index value of 48.80‒56.10%). Another previous study shows poor sustainability status for lowland rice farming in Merangin district [27]. In contrast, the study of Linda et al. [28] shows that the lowland rice farming in Denpasar had sustainable status.

The organic farming system can support rice farming sustainability from the ecological dimension. According to Ashari et al. [75], many people believe that organic farming is the best approach to achieving sustainable food production and resource use and that it is the best way to solve environmental degradation and farmers’ reliance on the agrochemical industry. In addition, organic farming reduces energy use and greenhouse emission by 40% compared to conventional ones [76]. However, it is less sustainable when viewed from the other four dimensions, as in the case of Tasikmalaya’s organic rice farming [77]. Hence, more environmentally friendly farms are likely to have lower technical efficiency [78].

#### Economic dimension

The sustainability status of increasing rice production in Bandung district, seen from the economic dimension, is categorically poor, with an index value of 35.25. This is closely related to the problem of farm labor that is increasingly scarce and difficult to obtain. The decreasing number of agricultural workers available has caused farmworkers’ wages to go higher, which consequently lowers the farmers’ income. It is thus necessary to start strengthening farmer business groups to create ways to potentially increase farmers’ income. A few possible ways can be through diversification of farming, integrated agriculture (a combination of agriculture and animal husbandry), appropriate machinery and equipment, and increasing economies of scale if possible.

In product marketing, most farmers sell their products to intermediaries because of the existing attachment of farmers’ capital to intermediaries related to the fulfillment of production inputs. Farmer’s business capital and intermediaries have been linked and established for a long time in rural areas. On the other hand, due to collateral requirements, formal bank credit available, such as the People’s Business Credit (KUR), is still difficult for most farmers to access. However, rice productivity that is relatively high, at an average of 6.35 tons/ha, and still has the potential to increase, manage to support the sustainability of rice production. Therefore, some applications of location-specific technology can be utilized in rice cultivation with close and direct guidance by field officers to encourage the farmers to do which can finally increase rice productivity.

Some other studies show larger index values, which mean better sustainability. Among the studies is Yusuf et al. [23], which reported that paddy rice farming in Siak district has a sustainability index value of more than 50%, from moderate to considerably sustainable for the region’s four subdistricts. In contrast, the study by Zuhdi et al. [26] in the same district revealed that rice farming is less sustainable from an economic dimension. Another study [79] demonstrated that rice farming with Integrated Pest Management (IPM) in Besur village, Lamongan district, was found to be sustainable from both the social and economic dimensions. It means that implementing IPM can be an appropriate way to increase the sustainability of rice farming in Indonesia.

#### Social dimension

The social sustainability status of increasing rice production in Bandung district falls into the less sustainable category with an index value of 30.79. This result is lower than that of the study by Yusuf et al. [24]. They reported that the sustainability of wetland rice farming in Siak district ranges from 47.70 to 56.70 (poor to moderate category). Such a low result of the social sustainability index for rice production in Bandung is especially because of the low educational level of the farmers, which is, on average, only managed to finish elementary school. Additionally, the age group is most farmers over 40 years old. This finding is quite in line with the study by Purba et al. [80] that mentioned an educational level significantly contributed to the rice farming sustainability in tidal swampland.

Millennials’ reluctance to enter the agricultural sector hinders farmer regeneration. It is common to see children from farming families reluctant to continue or carry on with their parent’s farm business [81]. This condition causes the slow adoption of agricultural technology by farmers. One of the government’s efforts to attract millennials to enter the agriculture sector is by establishing an Alsintan Service Business (UPJA). Through UPJA, it is hoped that millennial farmers will both manage rice fields and have other side businesses generated from UPJA. UPJA activities include rice milling and rice packaging to increase the selling value. Another effort to attract the millennial generation to enter the agricultural sector is by digitizing the agricultural sector (e-commerce, e-logistics). This millennial farmer group is an MSME business group engaged in the business sector.

#### Institutional dimension

The sustainability status of increasing rice production in Bandung district seen from the institutional dimension is in the less sustainable (poor) category with an index value of 48.11. The relatively low sustainability index value is due to the usage of chemical fertilizers and pesticides and the high rate of wetland conversion. Furthermore, the decreasing number of extension workers makes it impossible to reach the entire rice farming area, which will then impact the sustainability of rice farming. Meanwhile, Linda et al. [28] showed a higher sustainability index value (moderately sustainable) for rice farming in Subak Intaran Barat, Denpasar, for the same dimension.

#### Technological/infrastructure dimension

This dimension of technological/infrastructure has a sustainability index of 61.76, the highest score compared to other dimensions. This makes it fall into the category of moderately sustainable. This value of the sustainability index is slightly higher than paddy cultivation on marginal peat soil [21]. However, these values are both still in the same level of category.

This moderately technological sustainability category is due to technological advances that support rice farm development. The adoption of new VUB technology by farmers is relatively fast. It can be seen from the development of new varieties planted by farmers. The adoption of jajar legowo planting technology by rice farmers is relatively fast, as shown in the pattern of the j*ajar legowo* planting that has been applied in the region. It could be done efficiently by building a demonstration plot area as a pilot technology that farmers would then adopt. The application of rice postharvest technology has mainly been carried out traditionally using simple tools. Dryer and rice milling units are essential for postharvest activities as they can add value to the price of grain. The limited number of machinery can hinder the sustainability of rice farming in Bandung district.

Based on the results of these studies, the sustainability status of paddy fields and rice farming varies between regions but, multidimensionally, tends to be classified as less to moderately sustainable categories. Thus, in analyzing sustainability, it depends on the indicators used because, apart from those factors such as social, economic, technical, and institutional, including policies, sustainability is also determined by location-specific.

### Sensitive factors and key factors of a sustainable increase in rice production

In this section, the two group results of the leverage analysis presented are sensitive factors affecting sustainability and key (prospective) factors that have a dominant influence on the sustainable increase in rice production. MDS analyzed the determination of sensitive factors, while the MICMAC application was used to determine the key factors.

The determination of these sensitive and key factors is strategically essential in formulating a policy strategy to sustain rice production. Furthermore, leverage analysis is intended to see changes in the error value of determining the value of sustainability. The determination of sensitive attributes is based on the percentage change in the X-axis’s root mean square (RMS) ordination. The greater the value of the change in RMS, the more significant the role of these attributes in increasing the sustainability index [73].

#### Sensitive factors

**Fig 3:**
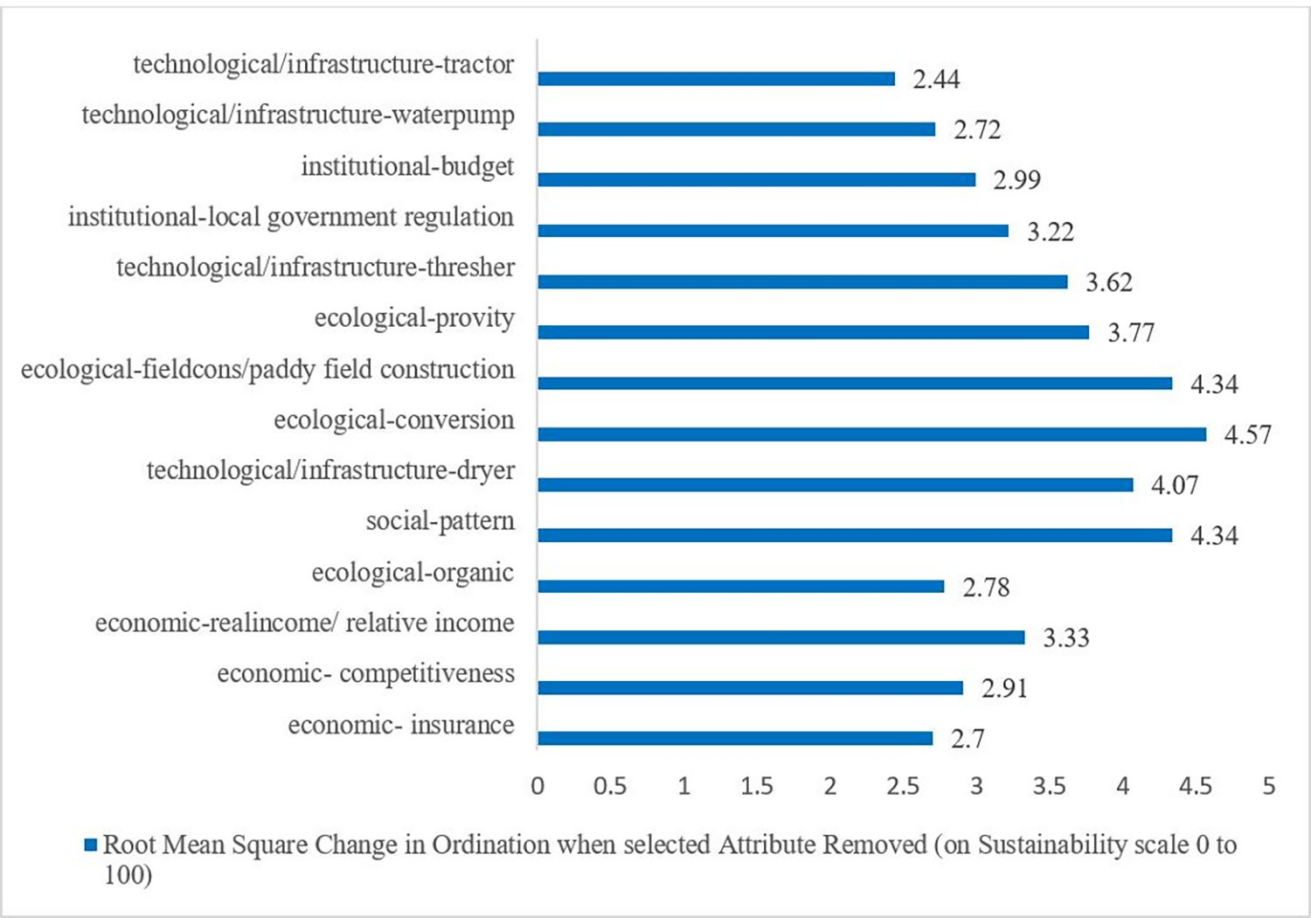
Leverage factors of rice production in Bandung district.

Based on the leverage analysis, of the 50 analyzed attributes (Table 1 and S1 Appendix), 14 of them sensitively affect the sustainability of rice production. Those 14 sensitive attributes consist of four ecological attributes, three economic attributes, one social attribute, two institutional attributes, and technological/infrastructure four attributes (Fig 3). In detail, each dimension can be explained as follows.

*Ecological dimension*. Of the 13 ecological dimension attributes analyzed (as can be seen in Table 1), four sensitive attributes affect rice production: the use of natural pesticides, land conversion, new paddy field construction, and rice productivity. Therefore, to increase rice production in Bandung district, it is necessary to prioritize these four attributes. Some previous studies support these findings, especially regarding land conversion [25], rice productivity, organic fertilizers and pesticides [22]. The other factors considered as sensitive factors of ecological aspect are land suitability [25] and land capability class [22].

*Economic dimension*. Of the 13 economic dimension attributes analyzed (as can be seen in Table 1), three sensitive attributes affect the sustainability of increasing rice production, namely the number of farmers participating in rice farming insurance (AUTP), the relative advantage of rice to other leading commodities, and the average income of farmers compared to the regional minimum wage. These three attributes/variables need some attention to support the sustainable increase in rice production. Meanwhile, Yusuf et al. [23] reported that the attributes of economic efficiency, availability of production facilities, and the marketing attributes of agricultural products were the sensitive factors in Siak district. Another study in the same region [26] found that business partnership is a leverage factor in the economic dimension. According to Frimawaty et al. [22], income proportion to total revenue, poor farmer proportion, farmer exchange value, government subsidies, production facility availability, comparative advantage, and rice prices were all found as the leverage factors for sustainable rice farming in Jambi in economic dimensions.

*Social dimension*. Of the 11 social dimension attributes (as seen in Table 1), just one sensitive attribute affects the increase in sustainable rice production in Bandung district, namely the pattern of farming management. Therefore, to improve the sustainability of the social dimension, the Bandung district government needs to ensure that farmers in their area manage rice farming well. Yusuf et al. [24] found different results, though. They found that training/counseling is the most significant attribute of the socio-cultural dimension that affects the sustainability of wetland rice farming in the Siak district. Another study [22] found farmers’ motivation and age, technology adoption rate, rice consumption growth, and government policy were the leverage factors affecting rice farming sustainability in Jambi province.

*Institutional dimension*. Of the five institutional attributes analyzed (as seen in Table 1), two sensitive attributes, namely the status of perennial land for rice and the proportion of local government budgets for the food crop subsector, affect the increase in sustainable rice production in Bandung district. Therefore, in order to increase rice production sustainably, these attributes need to be appropriately considered. Similarly, Barchia et al. [21] discovered that government support systems on agricultural input facilities provided a potential for rice cultivation sustainability. Meanwhile, farmer cooperative institutions are an attribute that needs to be addressed for the sustainability of rice farming in Bali [28].

*Technological/infrastructure dimension*. Of the eight technological/infrastructure attributes analyzed (as seen in Table 1), four sensitive attributes affect the sustainable increase in rice production, i.e., the number of rice dryers, the number of rice threshers, the number of water pumps, and the number of two-wheel and four-wheel tractors in the region. The four attributes of the technological dimension need attention to achieve a sustainable increase in rice production. Some other factors considered to be leverage factors for sustainable rice farming are, among others, postharvest technology, improved seed availability, the number of rice milling units, and pest management [22]. Apart from the number of rice threshers, the other study revealed that farm road is also crucial to support the sustainability of rice farming [28].

#### Key factors

**Fig 4:**
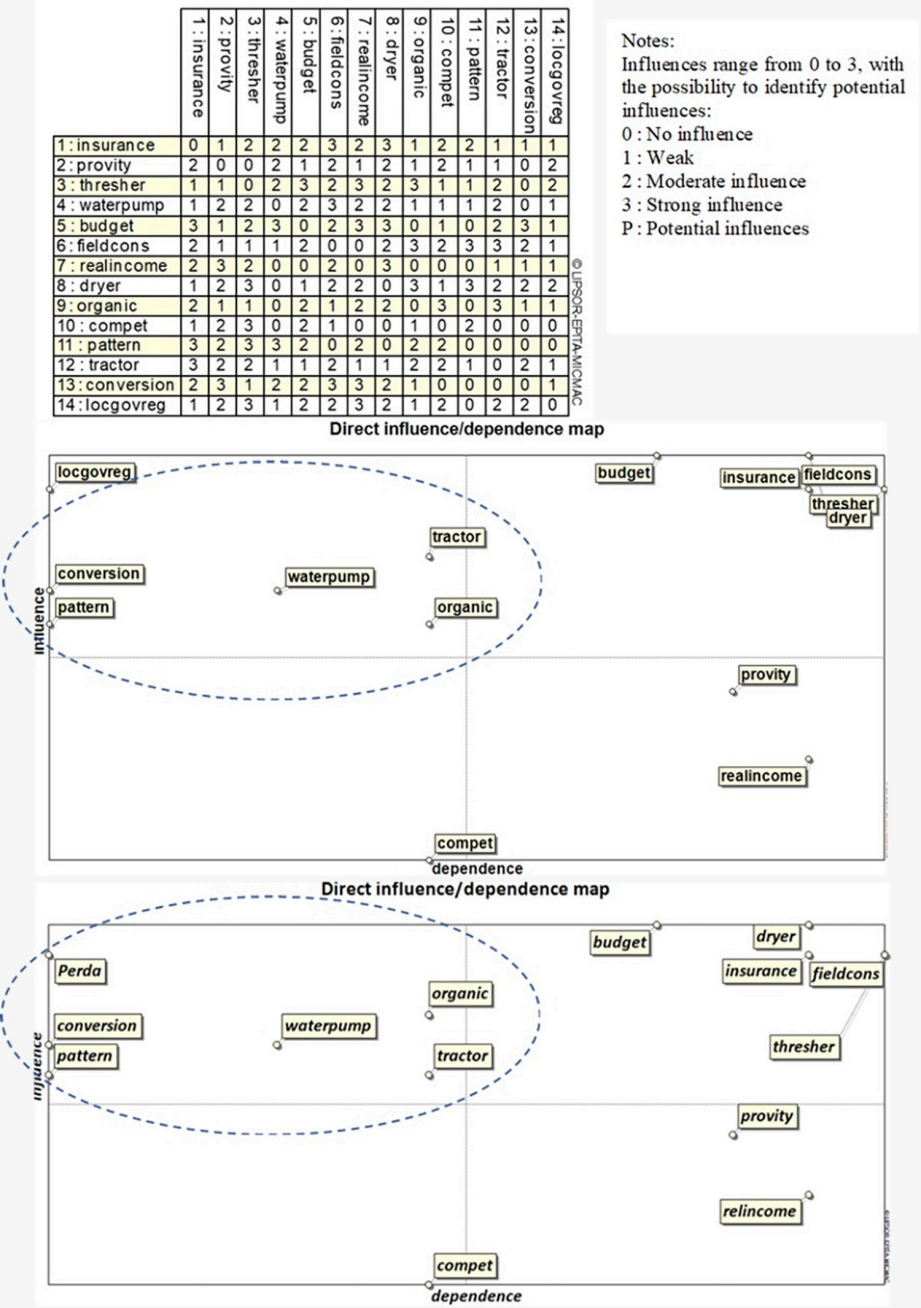
Key factors for a sustainable increase in rice production in Bandung district. Note:

Key factors are critical to the sustainability of rice production. These factors should then be prioritized in public policy to keep or even increase the sustainability of rice production [38]. Based on the results of the MICMAC analysis as presented in Fig 4, the key factors that influence the sustainability of rice production in Bandung district are as follows: (1) the perpetual status of land for rice, (2) the use of natural pesticides and fertilizers, (3) the number of water pumps, (4) land conversion, (5) rice farming management patterns, and (6) the number of two-wheel and four-wheel tractors.

**Table:** 

No.	Symbol	Description
1.	insurance	Percentage of farmers participating in rice farming insurance
2.	provity	Rice productivity
3.	fieldcons	Paddy field construction
4.	waterpump	Number of water pumps
5.	budget	Allocation of the local government budget for food crops sub-sector
6.	thresher	Number of rice threshers
7.	realincome	Farmers’ average income relative to the regional minimum wage
8.	dryer	Number of dryers
9.	tractor	Number of 2-wheel and 4-wheel tractors
10.	compet	The relative advantage of rice farming to other leading commodities
11.	pattern	Rice farming management pattern
12.	organic	Use of natural pesticides and fertilizers
13.	conversion	Land conversion
14.	locgovreg	Perpetual land status for rice

Regarding the status of perpetual land, the Bandung district government has already issued a regional regulation on Sustainable Food Agricultural Land (LP2B). However, there is no implementation of disincentives and incentives, so this regulation still needs to be revised and enforced to prevent the conversion of paddy fields to other uses. In Indonesia, it is estimated that the national rice field conversion rate was around 96,512 ha/year in the 2000‒2015 period [82]. In the case of land transfer from agricultural land to housing, law enforcement against violations of land-use change has not been carried out to the utmost extent possible. Although administrative procedures have been taken, criminals have reportedly never been prosecuted [83].

Chemical fertilizer and pesticide use are still high in Bandung district, but some farmers have obviously started reducing chemicals and gradually replacing them with organic materials. Indeed, it requires consolidating organic farmers under a solid organization to ensure the use of organic matter can effectively replace the chemical input. According to Latifi et al. [84], developing a suitable organizational structure is crucial in conservation agriculture growth. In the future, organic-based agriculture needs to be improved to maintain the sustainability of rice production in Bandung district. Besides to fulfill food security, such farming produces high-quality food that is good for people’s health while also being environmentally sustainable [85].

The availability of agricultural tools and machinery (*alsintan*) in Bandung district is still lacking, even though it is understood that mechanization is crucial to improve cost and time efficiency. With mechanization, the labor cost can be reduced, and the time needed for the farming process is shorter. For this reason, it is then essential to map the availability and needs of the *alsintan* following regional land conditions. However, Hidayah et al. [86] propose one specific thing that it is not just machinery to address sustainability issues. They argue that the application of adaptive technology suitable to local natural resources is indispensable in increasing rice farming productivity in rain-fed rice fields. A water pump, for example, is also vital for irrigation, especially during the dry season. With sufficient water needs, rice production will continue to be sustainable. Izar-Tenorio et al. [87] show that using electricity to pump irrigation water can boost agricultural output while improving financial sustainability.

Related to rice farming management, farmers generally carry out such business individually. Unfortunately, the land size of the farmers is relatively small, which makes rice farming not efficient [88]. Some studies show that land size significantly affects the technical efficiency of rice farming [80–82]. Therefore, it is necessary to organize business management through corporate-based rice farming management. Such management intends to strengthen the agricultural business system in single management, so there is an increase in production, productivity, quality, and potential added value. In its implementation, the management of corporate-based rice farming businesses must be carried out gradually and involve entrepreneurs in agriculture to cooperate with farmers. A participatory planning approach involving farmers is thus necessary.

## Conclusions

The sustainability status of increasing rice production in Bandung district is generally moderate. However, analysis by dimension shows variations in its sustainability status. The ecological and technological dimensions fall into a moderately sustainable category. In contrast, the sustainability statuses of the economic, social, and institutional dimensions are categorized as less sustainable. Out of 50 attributes, 14 are identified as sensitive attributes that affect the sustainability of rice production. The 14 attributes consist of four ecological attributes (the use of natural pesticides, land conversion, the new paddy field construction, rice productivity), three economic attributes (participation in rice farming insurance, the relative advantage of rice, the average farm income), one social attribute (the pattern of farming management), two institutional attributes (the status of perennial land for rice, the proportion of local government budgets), and four technological/infrastructure attributes (the number of rice dryers, the number of rice threshers, the number of water pumps, the number of two-wheel and four-wheel tractors). The sustainability of increasing rice production in Bandung district is influenced by six key factors: the status of perpetual land for rice, use of natural pesticides and fertilizers, land conversion, rice farming management patterns, the number of water pumps, and the number of two-wheel and four-wheel tractors.

Based on the results of this study, we recommend policies/programs to increase the sustainability of rice production as follows: implementation of the Regional Spatial Plan, promotion of the application of best practices of farming management, in particular, application of the organic fertilizers and pesticides, promotion of the use of agricultural machinery, and assistance for pre-harvest and postharvest production facilities, as well as farm financing. Future research should expand study sites to some other rice production centers with different attributes to enrich our understanding of the subject of rice sustainability.

## Supporting information

S1 Appendix Dimensions dan attributes used in analyzing sustainable rice production in Bandung district. (DOCX)

S1 Data Kite diagram rice sustainability. (XLSX)

S2 Data Rap-rice. (XLS)
