# Supplementary material for: Sustainability status, sensitive and key factors for increasing rice production: A case study in West Java, Indonesia
Source: PLoS One. 2022 Dec 30;17(12):e0274689. doi: 10.1371/journal.pone.0274689 (PMC9803313; doi:10.1371/journal.pone.0274689)
Supplement: S1 Appendix — (DOCX) [file pone.0274689.s001.docx]

**S1 Appendix. Dimensions dan attributes used in analyzing sustainable rice production in Bandung district.**

| **Dimension-factor/attribute** | **Score** | **Good** | **Bad** | **Description** | **Source of data** |
| --- | --- | --- | --- | --- | --- |
| 1. **Ecology Dimension** |  |  |  |  |  |
| 1. Use of chemical fertilizers | 0,1,2 | 0 | 2 | Average use of chemical fertilizers by farmers compared to recommendations in the district: (0) lower, (1) the same, (2) higher | Farmers, Office of Agricultural Services, BPS-Statistics |
| 1. Use of organic fertilizers | 0,1,2 | 0 | 2 | Average use of organic fertilizers by farmers compared to recommendations in the district: (0) lower, (1) the same, (2) higher | Farmers, Office of Agricultural Services, BPS-Statistics |
| 1. Use of chemical pesticides | 0,1,2 | 0 | 2 | Average use of chemical pesticides by farmers compared to recommendations in the district: (0) lower, (1) the same, (2) higher | Farmers, Office of Agricultural Services, BPS-Statistics |
| 1. Use of natural pesticides | 0,1,2 | 0 | 2 | In the last ten years, using natural pesticides: (0) lower, (1) relatively constant, (2) increasing | Farmers, Office of Agricultural Services, BPS-Statistics |
| 1. Total rainfall/year | 0,1,2,3 | 3 | 0 | Total rainfall compared to the average in the last ten years in the district: (0) low, (1) moderate, (2) high, (3) very high | BPS-Statistics, BMKG |
| 1. Number of dry months/year | 0,1,2,3 | 3 | 0 | Number of dry months compared to the average in the last ten years in the district: (0) low, (1) moderate, (2) high, (3) very high | BPS-Statistics |
| 1. Irrigation system | 0,1,2 | 2 | 0 | Irrigation system: (0) mainly using technical irrigation systems, (1) mainly using semi-technical irrigation, (2) mainly using simple systems | Farmers, Office of Agricultural Services, Office of Public Works |
| 1. Rice productivity | 0,1,2 | 2 | 0 | Rice productivity compared to the average productivity in the province: (0) lower, (1) equal, (2) higher | Farmers, BPS-Statistics |
| 1. Agricultural land conversion | 0,1,2 | 0 | 2 | Current land conversion rate compared to the average in the last ten years in the district: (0) decreased, (1) remained, (2) increased | Farmers, Office of Agricultural Services, Office of Public Works |
| 1. New paddy field construction | 0,1,2 | 2 | 0 | Current paddy field construction compared to the average in the last ten years in the district: (0) decreased, (1) remained, (2) increased | BPS-Statistics, Office of Agricultural Services |
| 1. Crop failure due to drought/flood/pests and plant diseases | 0,1,2 | 0 | 2 | Current crop failure compared to the average in the last ten years in the district: (0) decreased, (1) remained, (2) increased | Farmers, Office of Agricultural Services, BPS-Statistics |
| 1. Pressure of industrial and residential purpose on land use | 0,1,2,3 | 3 | 0 | Proximity to industrial and residential complexes: (0) very close, 0−0.5 km), (1) close, 0.5−1 km, (2) moderate, 1−1.5 km, (3) far, more than 2 km | Google map, farmers |
| 1. Utilization of rice field waste for soil fertility | 0,1,2,3 | 3 | 0 | Waste utilization: (0) no, (1) little, (2) moderate, (3) much | Farmers |
| 1. **Economic Dimension** |  |  |  |  |  |
| 1. Economic efficiency (RC)/ mean farmer income relative to the district's regional minimum wage | 0,1,2 | 2 | 0 | The average income of farmers relative to the minimum wage in the district: (0) low, (1) equal, (2) high | Farmers, Office of Agricultural Services, BPS-Statistics |
| 1. Changes in rice farming profits | 0,1,2 | 2 | 0 | Rice farming profits: (0) lower, (1) the same, (2) greater than the average of the last ten years | Farmers, Office of Agricultural Services, BPS-Statistics |
| 1. Rice production | 0,1,2,3,4 | 4 | 0 | Current rice production: (0) very low (1) low, (2) equal, (3) high, (4) very high compared to the average of the last ten years | BPS-Statistics, Office of Agricultural Services |
| 1. Farmer's exchange rate | 0,1,2 | 2 | 0 | Farmers' current exchange rate: (0) low, (1) the same, (2) higher than the last 10-year average | BPS-Statistics |
| 1. The price of rice at the farmer level | 0,1,2,3,4 | 4 | 0 | Price of harvested dry grain (GKP) / dry milled grain (GKG) at the farmer level: (0) very low, (1) low, (2) the same, (3) high, (4) very high compared to the average of the last ten years | BPS-Statistics, Food Security Agency |
| 1. Changes in real wages of farm workers | 0,1,2 | 2 | 0 | Changes in real wages of farmworkers: (0) low, (1) the same, (2) higher than the average of the last ten years | BPS-Statistics |
| 1. Percentage of smallholder farmers (<0.5 ha) | 0,1,2 | 0 | 2 | Percentage of smallholder farmers: (0) decreased, (1) remained, (2) increased compared to last ten years average | BPS-Statistics |
| 1. Number of agricultural workers | 0,1,2 | 2 | 0 | The number of agricultural workers: (0) decreased, (1) remained, (2) increased compared to the average of the last ten years | BPS-Statistics |
| 1. Access to capital for farming | 0,1,2 | 0 | 2 | Access to capital for farming: (0) easy, (1) difficult, (2) very difficult | Farmers |
| 1. Income from rice farming compared to total family income | 0,1,2,3 | 0 | 3 | Income from rice farming compared to total family income: (0) 100%, (1) 60-80%, (2) 40-60%, (3) 40% | Farmers |
| 1. Marketing agency that accommodates the harvest | 0,1,2,3 | 3 | 0 | Marketing institutions: (0) bondage system, (1) collectors/middlemen, (2) directly marketed, (3) through cooperatives/partners/STA | Farmers, Office of Agricultural Services |
| 1. The relative advantage of rice farming against other leading commodities | 0,1,2 | 2 | 0 | Rice profit: (0) lower, (1) equal, (2) higher than other commodities | Farmers, Office of Agricultural Services, BPS-Statistics |
| 1. Percentage of the number of farmers participating in the AUTP (Paddy Farming Business Insurance) | 0,1,2,3 | 3 | 0 | Percentage of the number of farmers participating in the AUTP (Paddy Farming Business Insurance) compared to the total number of farmers: (0) <25%, (1) 25−50%, (2) 51−75%, (3) >75% | Office of Agricultural Services |
| 1. **Social dimension** |  |  |  |  |  |
| 1. Proportion of rice farming HHs | 0,1,2 | 2 | 0 | Proportion of rice farming HHs compared to the total number of farmer HHs: (0) decreased, (1) the same, (2) increased compared to the average of the last ten years | BPS-Statistics |
| 1. Farmers’ formal education | 0,1,2,3,4 | 4 | 0 | Mostly: (0) Elementary school: (1) Junior high school, (2) Senior high school, (3) the largest percentage are high school graduates, (4) College | Farmers, BPS-Statistics |
| 1. Agricultural HHs’ participation in agricultural extension | 0,1,2,3 | 3 | 0 | Agricultural HHs’ participation in agricultural extension compared to the average for the last ten years: (0) never, (1) rarely, (2) often, (3) always | Farmers |
| 1. Motivation of farmers to do rice farming | 0,1,2 | 2 | 0 | The motivation of farmers to do rice farming; (0) decreased, (1) the same, (2) increased to the average of the last ten years | Farmers |
| 1. Frequency of paddy land use conflicts | 0,1,2 | 0 | 2 | Frequency of paddy land-use conflicts: (0) decreased, (1) the same, (2) increased to the average of the last ten years | Farmers |
| 1. Proportion of profit sharing | 0,1,2,3 | 3 | 0 | Proportion of cultivators: (0) <40%, (1) 40-50%, (2) 50-60%, (3) >60% | Farmers |
| 1. Family participation in rice farming activities | 0,1,2 | 2 | 0 | Family participation in rice farming activities: (0) only the head of the HH works in the fields, (1) the wife helps, (2) the wife and children help | Farmers |
| 1. Rice consumption | 0,1,2 | 2 | 0 | Consumption per capita: (0) decreased, (1) remained, (2) increased compared to the average of the last ten years | Farmers, BPS-Statistics |
| 1. Alternative business | 0,1,2 | 0 | 2 | Alternative business other than rice farming (other/non-agricultural commodities: (0) none, (1) agricultural/non-agricultural sector, (2) agricultural and non-agricultural sectors | Farmers |
| 1. Perceptions of the sustainability of rice farming | 0,1,2 | 2 | 0 | Perceptions of the sustainability of rice farming in ten years: (0) less sustainable, (1) relatively the same, (2) continuing | Farmers, Office of Agricultural Services |
| 1. Rice farming management pattern | 0,1,2 | 2 | 0 | Rice farming management pattern: (0) individual, (1) in a group, (2) corporation | Farmers, Office of Agricultural Services |
| **IV. Institutional dimension** |  |  |  |  |  |
| 1. Farmers' participation | 0,1,2 | 2 | 0 | Farmers' participation in farmer group's activities: (0) not active, (1) active, (2) very active | Farmer group, Office of Agricultural Services |
| 1. The presence of agricultural extension | 0,1,2,3 | 3 | 0 | The presence of agricultural extension: (0) never, (1) sometimes, (2) frequently, (3) very frequently | Farmers, Office of Agricultural Services |
| 1. Consistency of land use with RTRW (Regional Spatial Plan) | 0,1,2,3 | 3 | 0 | Suitability of land use with RTRW: (0) inconsistent <50%, (1) less consistent 50-70%, (2) consistent > 70% | Overlay map, GIS |
| 1. Perpetual land status for rice (local government regulation) | 0,1,2 | 2 | 0 | Perpetual land status for rice (local government regulation): (0) not yet decided, (1) in planning/discussion, (2) has been decided | Agency for Regional Development |
| 1. Proportion of local government budget for food crops sub-sector | 0,1,2 | 2 | 0 | The proportion of local government budget compared to the average in the last ten years: (0) lower, (1) the same, (2) higher | Agency for Regional Development |
| **V. Technological/infrastructure dimension** | | | | |  |
| 1. Number of tractors in the area (district) | 0,1,2,3,4 | 4 | 0 | Number of 2-wheel and 4-wheel tractors in the area (district) compared to the average in the last 10 years: (0) <25%, (1) 25-50%, (2) 51-75%, (3) 76-100, (4) >100% | Office of Agricultural Services |
| 1. Number of water pumps | 0,1,2,3,4 | 4 | 0 | Number of water pumps compared to the average in the last 10 years: (0) <25%, (1) 25-50%, (2) 51-75%, (3) 76-100, (4) >100% | Office of Agricultural Services |
| 1. Number of threshers | 0,1,2,3,4 | 4 | 0 | Number of threshers compared to the average in the last 10 years: (0) <25%, (1) 25-50%, (2) 51-75%, (3) 76-100, (4) >100% | Office of Agricultural Services |
| 1. Number of dryers dryers | 0,1,2,3,4 | 4 | 0 | Number of dryers dryers compared to the average in the last 10 years: (0) <25%, (1) 25-50%, (2) 51-75%, (3) 76-100, (4) >100% | Office of Agricultural Services |
| 1. Number of RMUs | 0,1,2,3,4 | 4 | 0 | Number of RMUs compared to the average in the last 10 years: (0) <25%, (1) 25-50%, (2) 51-75%, (3) 76-100, (4) >100% | Office of Agricultural Services |
| 1. Farmers’ adoption of new high-yielding varieties | 0,1,2 | 2 | 0 | Farmers’ adoption of new high-yielding varieties: (0) slow, (1) relatively fast, (2) very responsive | Farmer, Office of Agricultural Services |
| 1. Farmers’ adoption of the jajar legowo system | 0,1,2 | 2 | 0 | Farmers’ adoption of the jajar legowo system : (0) slow, (1) relatively fast, (2) very responsive | Farmer, Office of Agricultural Services |
| 1. Implementation of post-harvest technology | 0,1,2 | 2 | 0 | Implementation of post-harvest technology: (0) not implementing, (1) partly implementing, (2) implementing | Farmer, Office of Agricultural Services |
